# HIV Diagnoses Among Persons Aged 13–29 Years — United States, 2010–2014

**DOI:** 10.15585/mmwr.mm6707a2

**Published:** 2018-02-23

**Authors:** M. Cheryl Bañez Ocfemia, Richard Dunville, Tianchi Zhang, Lisa C. Barrios, Alexandra M. Oster

**Affiliations:** ^1^Division of HIV/AIDS Prevention, National Center for HIV/AIDS, Viral Hepatitis, STD, and TB Prevention, CDC; ^2^Division Of Adolescent and School Health, National Center for HIV/AIDS, Viral Hepatitis, STD, and TB Prevention, CDC.

In 2014, persons aged 13–29 years represented 23% of the U.S. population, yet accounted for 40% of diagnoses of human immunodeficiency virus (HIV) infection during the same year (*1*). During 2010–2014, the rates of diagnosis of HIV infection decreased among persons aged 15–19 years, were stable among persons aged 20–24 years, and increased among persons aged 25–29 years (*1*). However, these 5-year age groups encompass multiple developmental stages and potentially mask trends associated with the rapid psychosocial changes during adolescence through young adulthood. To better understand HIV infection among adolescents aged 13–17 years and young adults aged 18–29 years in the United States and identify ideal ages to target primary HIV prevention efforts, CDC analyzed data from the National HIV Surveillance System (NHSS)* using narrow age groups. During 2010–2014, rates of diagnosis of HIV infection per 100,000 population varied substantially among persons aged 13–15 years (0.7), 16–17 years (4.5), 18–19 years (16.5), and 20–21 years (28.6), and were higher, but less variable, among persons aged 22–23 years (34.0), 24–25 years (33.8), 26–27 years (31.3), and 28–29 years (28.7). In light of the remarkable increase in rates between ages 16–17, 18–19, and 20–21 years, and a recent study revealing that infection precedes diagnosis for young persons by an average of 2.7 years (*2*), these findings demonstrate the importance of targeting primary prevention efforts to persons aged <18 years and continuing through the period of elevated risk in their mid-twenties.

CDC analyzed data on persons aged 13–29 years who had HIV infection diagnosed during 2010–2014 and reported to NHSS through June 2016. Numbers and rates of diagnosed infections were calculated by year of diagnosis and by 2-year and 3-year age groups (ages 13–15, 16–17, 18–19, 20–21, 22–23, 24–25, 26–27, and 28–29 years) and selected characteristics. A single 3-year age group was required because of the odd number of years. All rates (per 100,000 population) were calculated using data from the U.S. Census Bureau for the denominators. Multiple imputation was used to assign a transmission category to persons reported without an identified risk factor (*3*). To assess trends during 2010–2014, the estimated annual percent change in HIV diagnosis rates was calculated using Poisson regression; changes were considered to be statistically significant if the 95% confidence interval (CI) excluded 0.

During 2010–2014, in 50 states and the District of Columbia, 78,337 persons aged 13–29 years had diagnosed HIV infection (Table 1). The overall HIV diagnosis rate was 21.3 per 100,000 population. By age group, HIV diagnosis rates varied substantially among persons aged 13–15 years (0.7), 16–17 years (4.5), 18–19 years (16.5), and 20–21 years (28.6). HIV diagnosis rates were higher, but less variable, among persons aged 22–23 years (34.0), 24–25 years (33.8), 26–27 years (31.3), and 28–29 years (28.7), with the highest rate in those aged 22–23 years (Table 1).

**TABLE 1:** Diagnoses of HIV infection* among persons aged 13–29 years, by year of diagnosis and age group — National HIV Surveillance System, United States, 2010–2014

Age group (yrs) at diagnosis	2010		2011		2012		2013		2014		2010–2014		
	No.	Rate^†^	No.	Rate^†^	No.	Rate^†^	No.	Rate^†^	No.	Rate^†^	No.	Rate^†^	EAPC^§^ (95% CI)
13–15	90	0.7	100	0.8	98	0.8	72	0.6	78	0.6	438	0.7	-5.9 (-12.0 to 0.5)
16–17	434	5.0	393	4.6	364	4.3	367	4.4	348	4.2	1,906	4.5	-4.0 (-7.0 to -0.9)^¶^
18–19	1,605	17.7	1,555	17.4	1,467	16.7	1,296	14.9	1,335	15.6	7,258	16.5	-4.0 (-5.6 to -2.4)^¶^
20–21	2,695	30.1	2,730	29.7	2,511	27.3	2,489	27.5	2,518	28.3	12,943	28.6	-2.0 (-3.2 to -0.8)^¶^
22–23	2,999	35.3	2,938	33.7	3,144	34.6	3,047	32.7	3,177	34.1	15,305	34.0	-0.9 (-2.1 to 0.2)
24–25	2,763	32.4	2,772	32.6	2,966	34.4	3,007	34.0	3,262	35.4	14,770	33.8	2.3 (1.1 to 3.4)^¶^
26–27	2,535	30.2	2,558	30.1	2,586	29.9	2,800	32.4	2,965	33.9	13,444	31.3	3.2 (2.0 to 4.4)^¶^
28–29	2,443	28.9	2,461	28.9	2,450	28.9	2,373	27.6	2,546	29.1	12,273	28.7	-0.3 (-1.5 to 1.0)
**Total**	**15,564**	**21.3**	**15,507**	**21.1**	**15,586**	**21.2**	**15,451**	**20.9**	**16,229**	**21.8**	**78,337**	**21.3**	**0.4(-0.1 to 0.9)**

**Abbreviations:** CI = confidence interval; EAPC = estimated annual percent change; HIV = human immunodeficiency virus.

*Data include persons with a diagnosis of HIV infection regardless of stage of disease at diagnosis.

^†^ Rates are per 100,000 population.

^§^ Trends were measured with EAPC in HIV diagnoses rates using Poisson regression.

^¶^ p<0.05.

Among persons aged 13–29 years with infection diagnosed during 2010–2014, blacks/African Americans accounted for the highest number and rate of HIV diagnoses (40,755 [52.0%]; 390.6 per 100,000 population), followed by Hispanics/Latinos (17,386 [22.2%]; 113.1) (Table 2). Among 66,471 males with diagnosed HIV infection, 59,634 (89.7%) had infections attributable to male-to-male sexual contact, and among these, males aged 22–23 years accounted for the highest number of diagnoses (12,275 [20.6%]). Among 11,866 females with diagnosed HIV infection, 10,462 (88.2%) had infections attributable to heterosexual contact, and among these, females aged 26–27 and 28–29 years accounted for the highest numbers of diagnoses (1,891 [18.1%] and 1,904 [18.2%], respectively). By region, the South accounted for the highest number and rate of HIV diagnoses among persons aged 13–29 years (40,667 [51.9%]; 148.2 per 100,000 population).

**TABLE 2:** Diagnoses of HIV infection* among persons aged 13–29 years, by age group at diagnosis and selected characteristics — National HIV Surveillance System, United States, 2010–2014

Characteristic	Total		Age group (yrs)															
			13–15		16–17		18–19		20–21		22–23		24–25		26–27		28–29	
	No.^†^ (%)	Rate^§^	No.^†^ (%)	Rate^§^	No.^†^ (%)	Rate^§^	No.^†^ (%)	Rate^§^	No.^†^ (%)	Rate^§^	No.^†^ (%)	Rate^§^	No.^†^ (%)	Rate^§^	No.^†^ (%)	Rate^§^	No.^†^ (%)	Rate^§^
**Sex**																		
Male	**66,471 (84.9)**	**176.8**	226 (0.3)	3.5	1,393 (2.1)	32.0	6,017 (9.1)	133.1	11,264 (16.9)	242.7	13,392 (20.1)	291.7	12,765 (19.2)	286.7	11,306 (17.0)	260.0	10,108 (15.2)	234.3
Female	**11,866 (15.1)**	**32.9**	212 (1.8)	3.5	513 (4.3)	12.4	1,241 (10.5)	29.0	1,679 (14.1)	38.0	1,913 (16.1)	43.5	2,005 (16.9)	46.7	2,138 (18.0)	50.5	2,165 (18.2)	51.0
**Race/Ethnicity**																		
American Indian/ Alaska Native	**323 (0.4)**	**51.5**	1 (0.3)	0.9	2 (0.6)	2.7	24 (7.4)	30.5	51 (15.8)	63.3	61 (18.9)	78.7	70 (21.7)	97.1	55 (17.0)	80.8	59 (18.3)	89.7
Asian	**1,333 (1.7)**	**34.7**	9 (0.7)	1.6	16 (1.2)	4.2	61 (4.6)	15.0	151 (11.3)	33.8	253 (19.0)	52.2	273 (20.5)	53.9	287 (21.5)	54.9	283 (21.2)	52.3
Black/African American	**40,755 (52.0)**	**390.6**	291 (0.7)	16.4	1,246 (3.1)	100.6	4,602 (11.3)	348.5	7,602 (18.7)	556.9	8,318 (20.4)	636.1	7,263 (17.8)	602.8	6,168 (15.1)	547.3	5,265 (12.9)	479.3
Hispanic/Latino^¶^	**17,386 (22.2)**	**113.1**	71 (0.4)	2.6	383 (2.2)	21.0	1,322 (7.6)	71.3	2,537 (14.6)	136.3	3,146 (18.1)	173.2	3,428 (19.7)	194.3	3,410 (19.6)	195.5	3,089 (17.8)	176.6
Native Hawaiian/ Other Pacific Islander	**92 (0.1)**	**60.3**	0 (0.0)	0.0	2 (2.2)	12.5	6 (6.5)	35.1	14 (15.2)	74.8	20 (21.7)	101.7	19 (20.7)	96.2	18 (19.6)	93.9	13 (14.1)	68.6
White	**15,419 (19.7)**	**37.2**	52 (0.3)	0.8	167 (1.1)	3.6	909 (5.9)	18.6	2,062 (13.4)	40.7	2,872 (18.6)	56.5	3,166 (20.5)	63.3	3,049 (19.8)	61.7	3,142 (20.4)	63.7
Multiple races	**3,029 (3.9)**	**166.6**	14 (0.5)	3.4	90 (3.0)	35.5	334 (11.0)	138.9	526 (17.4)	236.0	635 (21.0)	317.5	551 (18.2)	310.4	457 (15.1)	281.6	422 (13.9)	275.0
Transmission category**																		
**Male**																		
Male-to-male sexual contact	**59,634 (76.1)**	**—**	166 (0.3)	—	1,274 (2.1)	—	5,597 (9.4)	—	10,348 (17.4)	—	12,275 (20.6)	—	11,434 (19.2)	—	9,921 (16.6)	—	8,617 (14.4)	—
Injection drug use	**1,166 (1.5)**	**—**	4 (0.3)	—	11 (0.9)	—	56 (4.8)	—	141 (12.1)	—	183 (15.7)	—	226 (19.4)	—	273 (23.4)	—	273 (23.4)	—
Male-to-male sexual contact and injection drug use	**2,597 (3.3)**	**—**	8 (0.3)	—	40 (1.5)	—	171 (6.6)	—	389 (15.0)	—	453 (17.4)	—	542 (20.9)	—	481 (18.5)	—	513 (19.8)	—
Heterosexual contact**^††^**	**2,955 (3.8)**	**—**	13 (0.4)	—	55 (1.9)	—	177 (6.0)	—	373 (12.6)	—	468 (15.8)	—	550 (18.6)	—	622 (21.0)	—	698 (23.6)	—
Other^§§^	**120 (0.2)**	**—**	35 (29.2)	—	13 (10.8)	—	16 (13.3)	—	13 (10.8)	—	14 (11.7)	—	13 (10.8)	—	9 (7.5)	—	7 (5.8)	—
**Female**																		
Injection drug use	**1,262 (1.6)**	**—**	6 (0.5)	—	40 (3.2)	—	106 (8.4)	—	161 (12.8)	—	210 (16.6)	—	241 (19.1)	—	241 (19.1)	—	257 (20.4)	—
Heterosexual contact**^††^**	**10,462 (13.4)**	**—**	165 (1.6)	—	452 (4.3)	—	1,103 (10.5)	—	1,496 (14.3)	—	1,693 (16.2)	—	1,758 (16.8)	—	1,891 (18.1)	—	1,904 (18.2)	—
Other^§§^	**141 (0.2)**	**—**	40 (28.4)	—	21 (14.9)	—	32 (22.7)	—	22 (15.6)	—	10 (7.1)	—	6 (4.3)	—	6 (4.3)	—	4 (2.8)	—
**Region of residence**																		
Northeast	**12,812 (16.4)**	**99.9**	81 (0.6)	3.8	341 (2.7)	23.2	1,111 (8.7)	69.4	1,969 (15.4)	123.1	2,411 (18.8)	158.0	2,452 (19.1)	160.9	2,317 (18.1)	153.7	2,130 (16.6)	143.9
Midwest	**11,448 (14.6)**	**73.4**	55 (0.5)	2.0	311 (2.7)	16.8	1,262 (11.0)	66.1	2,069 (18.1)	105.5	2,303 (20.1)	122.7	2,053 (17.9)	115.6	1,821 (15.9)	104.0	1,574 (13.7)	89.2
South	**40,667 (51.9)**	**148.2**	240 (0.6)	5.1	1,061 (2.6)	33.9	3,958 (9.7)	122.2	6,975 (17.2)	207.5	8,129 (20.0)	240.7	7,570 (18.6)	231.2	6,645 (16.3)	208.0	6,089 (15.0)	191.1
West	**13,410 (17.1)**	**75.1**	62 (0.5)	2.1	193 (1.4)	9.5	927 (6.9)	45.1	1,930 (14.4)	90.4	2,462 (18.4)	111.5	2,695 (20.1)	124.1	2,661 (19.8)	125.0	2,480 (18.5)	116.4
**Total**	**78,337 (100.0)**	**106.3**	**438 (0.6)**	**3.5**	**1,906 (2.4)**	**22.5**	**7,258 (9.3)**	**82.4**	**12,943 (16.5)**	**142.9**	**15,305 (19.5)**	**170.2**	**14,770 (18.9)**	**168.9**	**13,444 (17.2)**	**156.7**	**12,273 (15.7)**	**143.4**

**Abbreviation:** HIV = human immunodeficiency virus.

*Data include persons with a diagnosis of HIV infection regardless of stage of disease at diagnosis.

^†^ Numbers <12 should be interpreted with caution.

^§^ Rates are per 100,000 population. Rates are not calculated by transmission category because of the lack of denominator data.

^¶^ Hispanics or Latinos might be of any race.

** Data statistically adjusted using multiple imputation techniques to account for missing transmission categories.

^††^ Heterosexual contact with a person known to have, or to be at high risk for, HIV infection.

^§§^ Includes persons with diagnosed infection attributed to hemophilia, blood transfusion, perinatal exposure, and risk factor not reported or not identified.

During 2010–2014, the overall HIV diagnosis rate among persons aged 13–29 years remained stable (estimated annual percent change = 0.4, 95% CI = −0.1 to 0.9) (Table 1). However, by age group, rates per 100,000 population increased during 2010–2014 among persons aged 24–25 years (from 32.4 to 35.4) and 26–27 years (from 30.2 to 33.9) and decreased among persons aged 16–17 years (from 5.0 to 4.2), 18–19 years (from 17.7 to 15.6), and 20–21 years (from 30.1 to 28.3) (Table 1) (Figure). Rates remained stable among persons aged 13–15, 22–23, and 28–29 years.

**FIGURE:**
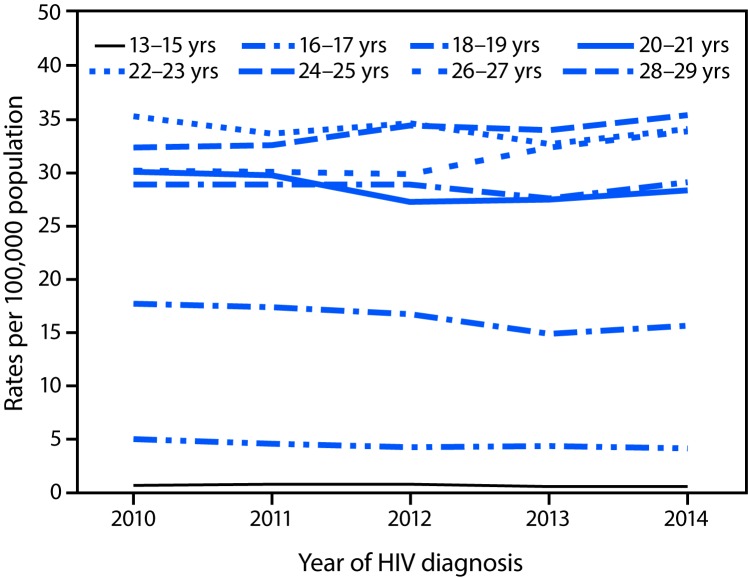
Rates* of diagnoses of HIV infection^^†^^ among persons aged 13–29 years, by year of diagnosis and age group — National HIV Surveillance System, United States, 2010–2014 **Abbreviation:** HIV = human immunodeficiency virus. * Rates are per 100,000 population. ^^†^^ Data include persons with a diagnosis of HIV infection regardless of stage of disease at diagnosis.

## Discussion

This analysis revealed large differences in rates of diagnosis of HIV infection with increasing age among persons aged 13–15, 16–17, 18–19, and 20–21 years. This report also documents trends in diagnoses during 2010–2014 by narrow age groups, with increasing rates observed among persons aged 24–25 and 26–27 years and decreasing rates among persons aged 16–17, 18–19, and 20–21 years.

Studies focused on adolescents and young adults with HIV infection commonly incorporate broader age ranges (e.g., 13–29 years), obscuring important distinctions that can contribute to a better understanding of HIV infections among persons during adolescence and into young adulthood (*4*). Adolescence and young adulthood are periods of considerable biologic and physiologic change and represent developmental phases when engagement in high-risk sexual behaviors and alcohol and other drug use peak and the risk for acquiring HIV infection increases (*4*,*5*). However, few HIV-related studies have taken into account these developmental transitions, and studies rarely include persons aged <18 years (*5*). A recent longitudinal study in an urban area with high HIV prevalence among men aged 16–20 years who have sex with men found that HIV incidence was just as high among participants aged <18 years as among older participants (*5*), highlighting the importance of including adolescents aged <18 years in research and prevention efforts, particularly HIV testing. A previous study has also shown delays in diagnosis of HIV infection of an average of 2.7 years in persons aged 13–24 years (*2*), indicating that the period of risk for HIV acquisition begins before age 18 years.

To help address the impact of HIV infection among adolescents and young adults, especially sexual and racial/ethnic minority populations, two national goals focus on persons aged 13–24 years as a priority population at risk to monitor the percentage of young gay and bisexual men who have engaged in HIV acquisition risk behaviors and the percentage of adolescents and young adults with diagnosed HIV infection who are virally suppressed (<200 HIV RNA copies/mL) through use of antiretroviral therapy (*6*). Unfortunately, adolescents and young adults are least likely to be linked to and retained in HIV care or to achieve viral suppression (*7*,*8*). In 2014, among men who have sex with men, who account for the majority of persons with HIV infection among persons aged 13–24 years, 48% were aware of their infection; awareness of infection is crucial to health and prevention (*9*). Among persons aged 13–24 years with infection diagnosed in 2014, 68% were linked to HIV medical care within 1 month of diagnosis, and among those living with diagnosed HIV infection at the end of 2013, 55% were retained in care, and 44% were virally suppressed (*8*). All of these indicators are well below national targets (*9*). Additional studies are needed to identify barriers that affect testing, retention in care, and access to health services, including the use of preexposure prophylaxis, among adolescents and young adults, particularly persons aged <18 years (*6*,*7*).

The findings in this report are subject to at least three limitations. First, the data presented reflect diagnoses of HIV infection, which are subject to diagnosis delay when compared with incidence, and are not necessarily representative of all persons with HIV infection. Whereas there are models available to estimate incidence, such approaches typically yield wide confidence intervals and unreliable estimates for narrow age groups. Second, trends in diagnoses of HIV infection might be attributed to changes in testing, transmission, or reporting. Finally, state laws affecting minors’ consent to care and disparities in access might also affect testing behaviors.

These findings underscore the importance of targeting primary prevention efforts to persons aged <18 years, specifically those aged 16–17 years, and continuing through the period of elevated risk in the mid-twenties. Much remains to be understood about the factors that affect adolescents and young adults at high risk for acquiring or transmitting HIV infection. CDC supports school districts and state education agencies that promote environments where teens can gain fundamental health knowledge and skills, establish healthy behaviors for a lifetime, connect to health services, and avoid becoming pregnant or infected with HIV or other sexually transmitted diseases (*10*). When implementing effective HIV prevention strategies, a multifaceted approach that incorporates the educational, social, policy, and health care systems can help support youths as they transition from adolescence into young adulthood (*7*).

**Summary**

**What is already known about this topic?**

In 2014, persons aged 13–29 years represented 23% of the U.S. population, yet accounted for 40% of diagnoses of human immunodeficiency virus (HIV) infection in the United States during the same year.

**What is added by this report?**

HIV diagnoses analyzed by age groups revealed striking differences in rates of diagnosis of HIV infection between ages 13–21 years. During 2010–2014, HIV infection diagnosis rates per 100,000 population varied substantially with increasing age among persons aged 13–15 years (0.7), 16–17 years (4.5), 18–19 years (16.5), and 20–21 years (28.6). HIV diagnosis rates were higher, but less variable, among persons aged 22–23 years (34.0), 24–25 years (33.8), 26–27 years (31.3), and 28–29 years (28.7).

**What are the implications for public health practice?**

The findings underscore the importance of using a multifaceted approach and targeting primary prevention efforts to persons aged <18 years and continuing through the period of elevated risk in their mid-twenties.

## Footnotes

* CDC’s National HIV Surveillance System (NHSS) is the primary source for monitoring HIV trends in the United States. Through NHSS, CDC collects, analyzes, and disseminates surveillance data on HIV infection.
