# *Erratum*: Vol. 67, No. 7

**DOI:** 10.15585/mmwr.mm6712a8

**Published:** 2018-03-30

In the report “HIV Diagnoses Among Persons Aged 13–29 Years — United States, 2010–2014,” on page 212, the second footnote for Table 1 should have read “^†^ Rates are **single-year rates** per 100,000 population. **Rates for 2010–2014 are comparable to single-year rates and are calculated as number of diagnoses during 2010–2014 divided by number of person-years at risk for diagnosis during 2010–2014 (x 100,000).**” On page 213, the third footnote for Table 2 should have read “^§^ Rates are **5-year cumulative rates** per 100,000 population. Rates are not calculated by transmission category because of the lack of denominator data.”

